# ‘Nonetheless biosocial’: experiences and embodied knowledge of birth cohort participants in the UK and Brazil

**DOI:** 10.1057/s41292-024-00344-z

**Published:** 2024-12-11

**Authors:** Rosie Mathers, Sahra Gibbon, Taylor Riley, Tatiane Muniz

**Affiliations:** 1 UCL, London, UK; 2 Anthropology Department, UCL, London, UK

**Keywords:** Birth cohorts, Biosocial, Embodiment, Intergenerational participation, UK and Brazil

## Abstract

The relative expansion of biosocial research within the life sciences has generated substantial interest from social sciences, with epigenetic science and scientists the primary target of critical commentary. This has led to a narrow perspective on what the biosocial is and how it is being (re)constituted within scientific research, highlighting a need to engage diverse publics in this unfolding terrain of knowledge making. Whilst birth cohorts are often a central resource and primary context for emerging fields of biosocial and epigenetic research, how cohort participants perceive and understand ‘biosocial’ interactions in the context of their lifelong and intergenerational participation is less well known. Drawing on pilot study research with birth cohort participants in the UK and Brazil, we comparatively examine how, in the absence of explicit references to a biosocial exemplar of epigenetics, biosocial dynamics are nonetheless understood by participants in relation to (i) embodied experiences, (ii) intergenerational participation, and (iii) understandings of the knowledge the studies aim to produce. Attending to different understandings of biological and social dynamics in diverse publics helps diversify and broaden the conceptual and methodological tools used to engage in and understand what the biosocial is and how it is coming into being.

This paper seeks to widen academic understanding and deployment of the ‘biosocial’ by extending its definition to include specific and situated public voices. Whilst the recent propulsion of biosocial theory has developed to deconstruct and reimagine how biology and society are treated within the life sciences, articulations of these dynamics have largely been reserved to academics alone. Within emerging biosocial paradigms it is important, however, to include multiple and lived viewpoints, to avoid constructing hegemonic ways of thinking, expand conceptual frameworks, and prevent the recurrence of colonial and eugenic abuses. We further suggest that the incorporation of diverse public perspectives, particularly among those who take part in studies generating biosocial knowledge, can help mitigate the extractive nature of research. To this end, we showcase how birth cohort participants understand the biosocial as a normal, quotidian, and intrinsic component of their everyday lives, a comprehension ultimately shaped by their lifelong research participation. By reflecting on their own memories and experiences of participation over many years and across generations, our interlocutors articulated the biosocial as an embodied experience wherein the artificial boundaries between the social and the biological somewhat dissolve. This recognition of the biosocial as an empirical, rather than academic, ontology repositions the biosocial as a fundamental phenomenon of ordinary life, an ‘inescapable metaphysics’ (Meloni et al. 2018, p. 1), in which our worlds and days are situated. This vantage point may help ground and galvanise researchers in the field to continue to find new ways to attend to the inherent symbiosis of the biosocial, rather than following methodological directions which seem to reconfigure the social as a covariate in public health analysis (see Gibbon et al. 2024).

Birth cohorts are long-term social and biological studies which follow participants from birth throughout their lives to capture data on population lifestyles and health at age or date defined intervals. They constitute one of the primary tools used by epidemiologists to track the mechanistic pathways to disease at a macro level, by enabling the measurement of varying social, economic, chemical, or otherwise harmful exposures and their associations with health outcomes. The birth cohort study is, therefore, an epicentre of biosocial research, providing a key site of interdisciplinary collaboration and discussion, wherein the parameters of what the biosocial is, and is not, are defined and deployed across multiple research fields. One of the most popular framings of biosocial knowledge within the last 20 years has been epigenetics, with a large number of high-profile international birth cohorts focused primarily on tracing the effects of early life environments on DNA methylation and child-to-adulthood phenotypic change, i.e. the UK’s Pace Consortium and the USA’s Nest Cohort (see Felix et al. 2018; King et al. 2015). Support from well-resourced global institutes has helped mobilise a cross-disciplinary interest in epigenetics, with recognisable examples including the DoHAD (Developmental Origins of Health and Disease) Society’s framework for researching the perinatal origins of disease, and the proliferation of EWAS (epigenome-wide association studies) databases, including EWASdb, EWAS Atlas, and EWAS datahub, and their use in various research agendas (Liu et al. 2019; Li et al. 2019; Xiong et al. 2020). Birth cohort studies, which carry a wide range of genetic data and social exposure variables, have been swept up in this ‘epigenetic moment’ as they provide a unique and profitable resource by which to investigate the impact of social environments on health biomarkers and population genetics. Whilst critiques of the limited treatment of social environments within biosocial research frameworks are beginning to emerge (Gibbon et al 2024; Neufcourt et al 2022; Vineis and Barouki 2022), the pervasive use of social factors as discrete variables, rather than as co-constitutive processes, remains the norm in public health models. Instead, owing to the widespread proliferation of epigenetic research, epigenetics has become the public ‘stand-in’ for biosocial knowledge across the media and other popularised domains.

Few studies have interviewed birth cohort participants’ themselves about their experiences of participation, despite the crucial role this situated public has in the creation and development of epigenetic and biosocial research. Whilst not an ethnographic study, Helen Pearson’s *The Life Project* (2016), which charts the history of Birth Cohort studies in the UK during a 70 year period, draws on over 150 interviews with birth cohort participants and, in so doing, provides a rare historical insight into how families were mobilised and incentivised to take part in intergenerational research. Other recent work has included important integrations of ethnographic and qualitative data in order to answer public health questions (e.g. Roberts 2021; Béhague and Gonçalves 2008), but has similarly remained limited when it comes to understanding participants’ experiences of research involvement itself. Finally, Rabinow’s (1992) work on biosociality, whilst similar in its framing of the importance of implicating public and social knowledge to understand biological phenomena, remains primarily focused on patient and genetic communities, rather than including persons whose experience is limited to research participation alone.

In this paper we draw on pilot study interviews with 14 paired mothers and daughters enrolled in the Pelotas Cohort study in Brazil and the ALSPAC (Avon Longitudinal Study of Parents and Children) study in the UK, to understand how participants make sense of their respective cohorts’ scientific agendas in relation to their own embodied experiences of research. These interviews were carried out as a pilot project and are the basis for the subsequent Wellcome Trust funded UCL Biosocial Lives of Birth Cohorts (BLBC),^1^ serving to establish an important foundation for the larger multinational comparative birth cohort study. Despite the current salience of epigenetics as a ‘stand-in’ for the biosocial, it is notable that only one participant, Alice, an original mother turned grandmother, mentioned it, and when she did so, it was as a self-confessed research novice:

[ALSPAC] were looking at… epigenetics? I don’t properly understand [laughing] all that stuff, but it was about whether my parents and grandparents had suffered from things like cancer, you know, what had been the causes of their death, whether they’d had long term illnesses? I can talk at length about my family history, I know quite a lot about it…

Alice’s comment speaks to two distinct forms of knowledge, the scientific proficiency of the study versus her own intimate grasp on her family history. In this paper we attempt to draw out these differing yet related forms of expertise to understand how participants view their own living histories in the light of long-term cohort participation. We suggest that, despite any claims to ignorance and not using the term, participants *nonetheless* have a strong and valuable comprehension of *biosocial* science, developed through their own intergenerational lives and embodied experiences taking part in the studies.

After outlining the current epigenetic landscape in order to situate our findings in the wider public domain, our analysis splits into four parts: first, an investigation into the motivations of cohort participants and the types of knowledge their participation affords; second, an examination of the way participatory knowledge is embodied; third, consideration of how intergenerational participation impacts biosocial understanding; and fourth, how these intersecting ‘on-the-ground’ knowledges compete with what participants see as the scientific ascendency of the study.

## Epigenetic epistemologies

Whilst the twentieth century was defined as the age ‘of the gene’ (Keller 2002), the beginnings of the twenty-first century have been characterised by an explosion of scientific and public interest in epigenetics (Haig 2012; Hurd 2010; Martens et al. 2011). This is reflected in the expanding number of publications, conferences, institutes, journals, media press, pop science books, and documentaries now dedicated to the topic (Meloni and Testa 2014: p. 432). Part of the allure of epigenetics is the novel epistemology it evokes, one in which biological determinism and predestination is replaced by physiological adaptability and flux. More exactly, epigenetics is the study of phenotypic changes to human genomes without modification or alteration of the original DNA sequence (Jablonka and Lamb 2006). It has been described in lay terms as a ‘flipping of a switch’ (Domann and Futscher 2004; Meymandi 2010) whereby exposures in the environment such as nutrition, pollution, or adverse social experiences ‘flip’ the original expression of a gene so that it adopts a new expression, introducing potential vulnerabilities, resiliences, or pathologies. Popularly attributed in its first usage by developmental biologist C. H. Waddington (1942) as a means of capturing the entire developmental process of the body, it has become a means of going above or beyond the limitations of the gene to invoke a powerful imaginary of plasticity and entanglement (Meloni and Testa 2014). Over the last decade, this epistemological current has formed a renewed interest in ‘biosocial’ sciences, whereby tracing the biological mechanisms and pathways through which social life ‘gets under the skin’ has become a central figure in epidemiological, anthropological, and interdisciplinary work (Ingold and Pálsson 2013; Kelly-Irving and Delpierre 2018; Krieger 2005; Meloni et al. 2018; Roberts 2021).

This epistemic shift has prompted a certain level of enthusiasm as well as scepticism, contestation, and debate from across the life sciences community (Haig 2012; Thayer and Non 2015). Whilst some have heralded the significant potential of epigenetics to resolve the entrenched dichotomies between the biological/social and nature/nurture debates through more synergistic research agendas and methods (Dubois et al. 2020), others have warned against the ways in which hegemonic biocentric logic continues to play out within epigenetic research, such as the increasing ‘digitalization’ of the environment and the preference for ‘hard’ biomarkers over ‘soft’ measures of human lived experience (Niewöhner 2011; Keller 2010; Lloyd and Müller 2018; Rapp 2018). Similar concerns exist among academics engaged with biosocial research that epigenetics merely remains a rebranded mechanism for parsing the social as a conduit for the biological (Meloni et al. 2018; Meloni 2016) or reconfiguring genetic determinism through “causal enclosures” (Roberts 2022). To counter debates surrounding the “epistemic ostentatiousness” of viewing epigenetics as scientific exactitude (Pickersgill 2016), social scientists have begun to look for the local contexts in which epigenetic and biosocial epistemologies are made (Lloyd and Raikhel 2018). By applying biosocial logic to different local contexts, new emic knowledges about how biological and social life interact are developed (Gibbon 2018), spawning novel and situated understandings of biology (Niewöhner and Lock 2018).

## Mediating epigenetics in public domains

There remain, however, many questions about how diverse publics respond to the different trajectories in which new, unfolding and often ambiguous articulations of the biosocial are articulated. Because of its compelling new image of human biology as a-work-in-progress, epigenetics has arguably become the poster child of biosocial science, widely captivating the public, media and academic imagination.^2^ Epigenetics has, therefore, been termed the “window”, or frame, through which diverse publics currently encounter biosocial theory (Dubois et al. 2020, p. 3). As Dubois et al. (2019) have noted, one dominant theme in the media includes an intense focus on the scientific ideas of transgenerational transmission, particularly in relation to areas concerning trauma, nutrition, or cancer risk. Importantly, these yet to be proven pathways of epigenetic transmission are often framed through the idea of ‘individual control’ and ‘empowerment’ that are, as others have shown, highly gendered (Lappé 2016). Indeed, the focus on maternal effects, in relation to nutrition and early development, with the consequent targeting of the behaviour of pregnant women and their future offspring, has been subject to intense critical reflection by social scientists, whilst also leading to growing interdisciplinary awareness of this problem (Richardson et al. 2014; Kenney and Müller 2017; Warin and Hammarström, 2018). Another significant and novel aspect of this discourse is the ‘scientization’ of epigenetics where specific scientific terms such as ‘methylation’ are used in the absence of full explanation (cf Stelmach and Nerlich 2015). Whilst this lends credibility to certain publicly made claims, it also means that correlations and causations are conflated, enabling vague or ambiguous associations to become concretised and used to support still emerging scientific knowledge about how environment and behaviours interact with health.

Whilst these studies of media discourse are an important step towards further understanding how epigenetics and biosocial dynamics are circulated and disseminated in public contexts, there is a need for further examination of these developments within specific public groups. Thus far there have been important contributions focusing on the uptake of epigenetic science and knowledge in indigenous communities in Australia (Warin et al. 2020; Keaney et al. 2023), in US educational settings (Müller and Kenney 2021) and in the application and dissemination of DOHaD policies in South Africa (Pentecost 2024). Here, we seek to contribute to these analyses by examining how particular publics, namely participants involved in intergenerational birth cohort studies, express in both direct and indirect ways how they understand the interactions between the biological and social. In contrast to previous work, we argue that such communities are not simply situated recipients of new epigenetic science and knowledge, but come to intimately embody and know about the biosocial through the quotidian contexts and consequences of research participation.

## Methods

Our data come from two pilot studies carried out by Rosie Mathers and Sahra Gibbon, and two research assistants in Brazil,^3^ who worked with ALSPAC and the Pelotas Cohort Study. This pilot work was the starting point for a wider study exploring how ethnography can be used in longitudinal cohort studies and comparing the resulting data across four cohorts in the UK, Brazil, Portugal, and the Netherlands. Because of the relatively small scale of the pilot project, recruitment consisted of seven family pairs, totalling eight respondents in England and six in Brazil. Recruitment materials were sent out to original study participants who had children of their own and who, in England, lived within a 15-mile radius of the Bristol cohort, and in Brazil, were born in the urban area of Pelotas. Because of limitations in terms of sample size and the filtration of socio-demographic characteristics, we were not able to recruit intentionally for diversity within the sample. Respondents all identified as women, reflecting the typical gender imbalance within birth cohorts of attrition primarily affecting men (see e.g. Boyd et al. 2012 re: ALSPAC). This paper, therefore, does not claim generalisability for the cohorts, but seeks to highlight the common threads and insights found in the analysis of this small but rich data sample.

Semi-structured interviews were designed to examine the factors influencing the decision to participate in a birth cohort study, how cohort participants perceive and experience multi-generational research, and how biosocial research is understood in relation to being both a cohort participant and mother/grandmother. Mathers and Gibbon each carried out four interviews with ALSPAC participants and research assistants Helena Fietz and Valéria Aydos completed three interviews with participants from the Pelotas Cohort Study. In addition, Taylor Riley and Tatiane Muniz, who are working on the wider BLBC study with the ALSPAC and Pelotas cohort, respectively, contributed to data analysis and the writing of this paper. Ethical approval was obtained from the ALSPAC Law and Ethics Committee, the Ethics committee at the Federal University of Pelotas and the UCL Research Ethics Committee.

ALSPAC is a long-standing regional birth cohort based in the city of Bristol in the UK. Pregnant women resident in the former county Avon, with expected dates of delivery between 1st April 1991 and 31st December 1992, were invited to take part in a study that initially enrolled 14,541 women and 13,988 children.^4^ The study is effectively multi-generational as it has now recruited over 3 generations of parents and children. The Pelotas Birth Cohort Study is also a long-standing birth cohort study based in the city of Pelotas in Southern Brazil. The first wave in 1982 recruited nearly all pregnant women who gave birth that year (*n* = 5914) with subsequent studies initiated locally every 11 years, meaning there are approximately four linked birth cohort studies in a city with a population of 325,689. The original Pelotas cohort is not explicitly multi-generational but there are now more than two subsequent generations involved and linked to the 1993 study wave. When it comes to receiving study results, neither cohort provides specific feedback on health data to participants, although participants in both cohorts may receive health information indirectly in the process of routine cohort evaluations and data collections. At ALSPAC, participants will either be provided a letter for their general practitioner (GP) or may be contacted to have further testing done by their GP or the local hospital, if obtained results are outside of a specified range. In Pelotas, participants may also receive informal feedback during evaluations such as in-the-moment reports generated by spirometry equipment, DEXA scans, and body fat percentage monitors (BODPOD), although these are provided with the caveat that they do not hold the value of a full medical report.

The pilot study research that informs this paper was undertaken between November 2019 and August 2020. We recruited and undertook joint interviews with four pairs of adult participants in Bristol and three pairs of adult participants in Pelotas. This included parents from the first wave of recruitment and their now adult children who, in all but one case, had children involved in cohort research in some form. All participants were given pseudonyms and are referred to either as original mothers/grandmothers or original children/adult daughters. Half of the interviews were undertaken in participants’ homes in Bristol, with the other half online, and all interviews in Brazil took place online, due to Covid19 restrictions. Whilst our study sample is relatively small, we highlight several key differences between cultural contexts, enabling us to suggest that future comparisons between birth cohorts in diverse locations will likely elucidate specific iterations of biosocial knowledge; a project built on by the BLBC initiative led by Sahra Gibbon. The possibilities for comparative work on birth cohorts between the minority world of high-income countries and the majority world of middle- and low-income countries not only offers scope to address culturally relative perceptions of the body, health, and illness, but to further understand how such paradigms come to formulate culturally specific models of biosocial science. Such comparative participation-based work, begun in earnest with this pilot, will contribute to wider efforts to localise biosocial knowledge (Niewöhner and Lock 2018) and provide contextually relevant frameworks for public health and social determinants models.

### Contextualising differences between participant motivations and knowledge in the Pelotas and ALSPAC birth cohorts

Whilst both ALSPAC and Pelotas cohort participants recognised that the studies they participated in were for the purposes of research and were a way of contributing to scientific knowledge, there were significant differences between how these broad understandings were situated. This in turn diversely shaped motivations for participation and, to some extent, their conceptions of the biosocial knowledge the studies were aiming to produce. Before talking more directly about how the biosocial was nonetheless articulated by participants, we first outline some of the contextual differences regarding participants’ motivations and understandings around taking part.

For those Mathers and Gibbon met in the ALSPAC cohort, there was a strong sense that the research they were involved in contributed to a specifically ‘collective’ understanding of health and development over time, and that the motivation for participation was located less in any immediate personal benefits and more in terms of a wider collective purpose. This was articulated in terms of an undefined community—‘them’—that many participants perceived as the research beneficiaries. For instance, Sue, an original mother turned grandmother, described “doing that bit to help” and how the cohort had enabled the researchers “to find out so much” for future generations. For her, there was sense of pride in being part of this wider collective effort, as she put it: “All these people …who are doing Children of the 90’s [the participant-facing name of ALSPAC] I’m just so pleased that we’re part of that, to put in that to help [a] better future… with other people and kids”. For original mother Alice, who was also recruited whilst pregnant into the study, her involvement had generated real excitement which she saw in terms of an “experiment… something that was gonna really make a difference”. She also talked about how being involved aligned with her being “a socialist” and that “people have actually got to put the effort into to make it [the research] work”. This sentiment was echoed in the feelings of her now adult daughter Lauren who framed her involvement as “doing your bit to contribute to something that you may or may not ever reap any benefit [from]”.

For some of the participant pairs our team met in the Pelotas 1993 cohort study, there was a similar sense of contributing to a collective goal, with a few of those we met talking about how their involvement was ‘advancing’ knowledge. However, this was often very vaguely articulated, or, in contrast with ALSPAC participants, not always expressed as a motivation for participation. For Ana and her daughter Vivian there was a feeling of indifference about their participation and a lack of clarity around the aims of the study, whilst they further highlighted a sense of personal benefit to being involved. As Ana said:

I never really quite understood the basis of it (*bem o fundamento, assim*)... it’s research data, kind of to get a future idea, about development, maybe, you know, for people’s lives like that, something that doesn’t help but doesn’t get in the way either (*não atrapalhar e nada*) […] In a way, it has brought some benefits, right, with the examinations, and that is great. I think so, I always thought it was nice to participate.

For others who we were interviewed from the Pelotas Cohort Study it was clear that there were specific and readily identifiable personal benefits to participation, especially the regular access to routine health care that was otherwise harder to obtain through the Brazilian public health care system or SUS (*Sistema Único de Saúde*). Original mother Fernanda commented participation was “good for her to get access to doctors and stuff”, describing participation through the word *acompanhamento*, which refers to a regular and routinised form of health check-ups in Brazil. Raquel, Fernanda’s daughter, also pointed out that “for me it was great, because I felt looked out for (*controlada assim*)… they always made it very clear that any result or change (*alteração*) in the exams they would warn you, so I felt safe”. Initially she talked specifically about the number of ultrasounds she had received as a participant, pointing out that “in this way, we somehow feel a little bit more remembered” by the healthcare system. She went on to reflect that her brother who had not been included in the study had experienced less access to health care services and how, in contrast, her son had been able to access certain kinds of care more easily because of his involvement with cohort research. This ranged from more trivial things, such as the cohort providing her son’s first trip to the dentist, to the more profound, including her son’s diagnosis of autism which Raquel attributed directly to his involvement with Pelotas Cohort, saying, “if there were no such study we wouldn’t know and wouldn’t have this understanding”.

Interestingly, ‘socialist’ ALSPAC grandmother Alice also found that her involvement in the study provided useful information on one of her children who had experienced birth trauma, adding that the tests he underwent at a young age proved to be “very useful”. One test, in particular, was memorable for its efficacy:

They’d show you a face and you had to say whether it was a happy face or—you know a cross face and so on, and it became apparent that he [her son] wasn’t very good at telling the difference… he was like 4, 5 years old and he couldn’t differentiate between a smiling face and a—a frowning face.

Alice further reflected that her son had a hearing problem which she thought might have been detected by ALSPAC tests, although she couldn’t fully remember if this was the case. Given her initial motivations for joining the study, it seemed as though Alice felt these examinations were a benefit, but not the primary reason for her ongoing contribution. Crucially, she did not attribute her knowledge of her son’s condition solely to the study, not always remembering the extent of its utility, in the same way that Raquel pinpointed, suggesting less reliance on the personal health findings afforded through her participation.

Participants’ comments, therefore, pointed to differing motivations across international contexts, with those from the UK generally incentivised by being part of a ‘bigger picture’ whilst those from Brazil, who whilst at times shared this view, more often referenced improved access to healthcare as a reason for their sustained involvement. These varying motivations, to some degree, afforded participants with differing understandings of what the biosocial is, with some participants interpreting their studies’ biosocial aims and methods primarily as a sort of open-ended exploration for generating new public health findings, whilst others felt them to be more closely linked with direct health improvements for resource-lacking communities. As we will see in the final section, these connected but divergent viewpoints influenced the degree to which participants valued their own knowledge. Those who saw the research as an investigatory process felt less comfortable declaring themselves knowledgeable on seemingly undefined subjects, whereas those who saw the research as having tangible outcomes for their own families were more confident in articulating specific and personal examples of the biosocial.

### Embodied experience

During interviews, participants articulated their involvement in the cohorts as embodied in many respects. From the somatic experience of a body under scrutiny, to multisensory engagement with technologies that generate biomedical knowledge, and the daily realities of tracking one’s physiological health functioning, research participation affords those who undergo it with new and intimate ways of relating to their own bodies. Whilst these experiences do not necessarily extend beyond their research engagement, we contend that this dimension of involvement provides participants with a unique vantage point through which to reflect on the entangled nature of their biological and social lives.

For participants, the clinical dimension of being in a cohort study is frequently highlighted by their atypical access to specialised biomedical monitoring techniques. During interviews, such technological interventions were often described by participants as being the most intriguing, exciting, and memorable. As original Pelotas daughter Carla remembered:

They took blood, I used a pump (*bombinha*) too, and we got into a machine too, very sinister, which looked like an egg, I forgot the name of this machine... I think it calculated the... body fat index, or was muscle mass, something like that, it was pretty cool.

By engaging with novel technologies, which produce new images, data, and associated meanings about physical health, participants were presented with fresh perspectives on their bodies that departed from the everyday. Visualisation techniques such as bone density (DEXA or DXA) scans, whilst not uncommon in specialised clinical settings, felt strange, futuristic, and fun for participants, and several mentioned the material satisfaction of being able to keep copies of their own scans. These prized technological artefacts were shared with family members, displayed on the fridge, or now, having been lost, were desired to be found and viewed again.

Furthermore, during interviews, the ability to recall certain research activities seemed moderated by participants’ sensorial engagement with unusual technological machinery, meaning not only were the experiences of participation embodied but so were the resulting memories. When asked to reflect on their time in the study, participants varyingly referred to their experiences of collecting clinical data as enjoyable and pleasurable, and at other times, tiring or uncomfortable. Each return for a clinical follow-up was often permeated by a specific set of sensations such as excitement, fear, curiosity, or sometimes boredom. These emotional responses related to several factors, including whether participants were enthusiastic or nervous to find out how their health had evolved since the last clinic visit, or whether old and perhaps tedious examinations were being run, or new and potentially anxiety-inducing tests were being introduced.

Some embodied experiences were very vivid and memorable, such as one reported by Vivian from Pelotas, who described wearing an accelerometer, a device used to measure the intensity and frequency of physical activity, as almost supernatural. As a 10-year-old child she had associated the device with a popular cartoon character called ‘Ben 10’ who was known for wearing a magic watch:

I had to use a watch that monitored—I think it was the heartbeats, I don’t know if it was the calories I spent, how much I walked, everything that I made that watch monitored, so, in my organism like, this—my metabolism, these things like that, and I don’t know how long I used it, but it was a long time, I think a week or so. I had to wear that watch, and I couldn’t take it off for nothing… even to take a bath. I know everyone said it was the clock of Ben 10… [laughs] it was really funny…

Vivian’s comments frame her participation as something special and interesting, but also highlight her recognition of the biosocial nature of her self-generated data. In discussing the accelerometer, Vivian seems to indiscriminately integrate her biological processes, such as her heart rate and metabolism, with her social experiences, such as her eating habits and the distance she walked that week, and the ways in which they mutually inform each other. In her recollection, the boundaries between the biological and social spheres somewhat dissolve, so that ‘everything that [she] made’, or did, that week is treated as having equal value. The highly embodied nature of the ‘Ben-10 watch’ made it a memorable example which, during her interview, enabled Vivian to recall the two ‘bio’ and ‘social’ aspects of her life concurrently. Noting her own metabolising organism as materially engaging with the world around her, Vivian recognised herself as both a social and biological being, a perspective which is perhaps both uniquely situated and, on another level, very normal.

In the UK, original study child Jackie, whose young son is now the third generation enrolled in ALSPAC, reflected that during physical procedures, the study was always amenable to making participants feel as comfortable as possible. Her toddler son’s fear of needles, for example, inhibited his ability to take part in routine blood collections, which is a boundary always respected by the study. Jackie went on to describe how her visits with her son contributed to a growing awareness of his body and behaviours as objects of scientific inquiry. This informed her rationalisation for why the study kept involving new generations, so as to capture children’s developmental milestones over time:

Some of the things [tests] he couldn’t do… [last time] and then today he flew through [them]—which was nice, cause you notice that improvement then, which you might not see on a day to day sort of basis… [and] obviously if you compare say for example *my son’s* study this morning to *my* study when I was four years old… could I do the colours, and the counting like he could, or did that take me an extra year?

Participating in the study gave Jackie the chance to step back and see how the documentation of her son’s behaviours and habits, alongside records kept about his physical development which she also referenced, created a holistic picture of his overall health. Her memories of participation coupled with her son’s embodied activities further enabled Jackie to rationalise the cohort’s use of multiple generations as a biosocial practice tallying the effects different environments have on early-year development.

In this way, participants’ long-term involvement in birth cohorts granted them an embodied understanding of the biosocial passing of time, both through the biological and social documentation of the ageing process. Sue, Jackie’s mother, reflected on the implications of her ageing body on research, saying, “as you get older, I’ve been told recently that your veins shrink—so they’ve got to use a small needle on me otherwise I don’t go in, it don’t get nothing out”. Similarly, Jackie’s memory of being involved in sub-studies at specific time-points over the lifecourse, such as the ‘Turning 24’ study, suggested to her that as time progresses, new questions and variables indicative of certain life stages are incorporated into the study. Sue and Jackie’s intimate understanding that bodies evolve over the lifecourse, exemplified by Jackie’s son’s counting success or Sue’s shrinking veins, as well as their recognition that ALSPAC researchers are working to capture these processes, helped reframe everyday ageing to them as valuable scientific data. Embodied engagement with the study emerged into the narratives and understandings both women developed around the study, including a focus on specific practices such as blood collection, which they thought likely to be particularly generative.

Participation in the study also has the potential to subvert expectations about how and when aged bodies are subjected to and surveyed by certain technological equipment. As original daughter Lucy said:

Most of the time I quite enjoyed doing things that *ordinarily* someone my age wouldn’t get to do, like a heart scan—you wouldn’t normally get to sit there and—and look at a scan of your heart and take pictures and ask questions and—I quite liked all that kind of stuff because why else would you normally scan an 18 year olds’ heart unless there was something severely wrong! [laughs].

Like Carla, the exposure to seemingly ‘sci-fi’ technology at a young age afforded Lucy a novel and unforgettable way to relate to her own physicality, one which she could readily call to mind many years later. Such encounters are often particularly affecting, and provide rare instances in which participants get to see what is ‘under their own skin’ and recognise themselves as functioning physiological organisms. During discussion of these memorable experiences, participants appear to be confronted with the biosocial nature of their own bodies and lives, as their experience of the artificial boundaries between their biological and social selves appears to disband. Furthermore, birth cohort participants’ focused attention to the embodied passage of time and developmental processes of ageing helps to reinforce the biosocial as an essential and everyday fact of their intergenerational lives.

### Inheriting birth cohort participation

Just as families pass their genetics down through the generations, our interlocutors described a similar form of social ‘heritability’ present in cohort recruitment and participation practices. Uptake into the relative birth cohorts was handed down from parent to child to grandchild, seemingly based, at least in part, on the extent to which families had positive social experiences and good personal relationships with the study. In turn, this process of social heritability highlighted to participants their studies’ capacities for capturing an ever-evolving socio-cultural context. Both ALSPAC and Pelotas participants saw their intergenerational involvement as providing a kind of contemporary history, reflecting, for example, recent changes in human lifestyles, health-related social behaviours, or new emerging medical practices and technologies, within the last 30 or so years. Participation thus creates a historical snapshot of the overarching structures and associated exposures prescient in each generation, rendering participants’ biomedical data as products of moments in time, and therefore, recasting participants’ knowledge of their own bodies, health, and biology as firmly rooted in the social.

For 1993 Pelotas cohort participant, Raquel, the purpose of the study was “to see how we change (*a ver nossa evolução*)… and to compare with other generations”. Raquel reflected a general feeling among all participants who saw their lives evolving in tandem with the developing social and historical milieu in which they live. One example mentioned during interviews was the way clinical diagnoses have changed from one generation to the next. Reflecting on her son’s autism diagnosis, Raquel commented, “autism in my childhood was something that was not heard of” adding that potentially several new conditions were now being discovered, “autism is one thing [condition], but there are several things [conditions], there was the microcephaly wave a few years ago, wasn’t there?” This allusion to diagnostic trends hints at the temporal changes inherent in long-term research participation and an ever-changing social environment which, in turn, ushers in novel consequences for those involved.

Several ALSPAC pairs discussed this through the lens of childhood home environments. Lauren, Alice’s adult daughter, reflected: “Your upbringing [meaning Alice] like, your parent’s upbringing would obviously affect your upbringing, so why wouldn’t they ask about environment and that sort of thing”. Such appreciations that childhood environments impact adults in later life were frequently articulated by family members who, discussing these realities in mother-daughter pairs, could see how ancestral habits and behaviours had trickled down into and shaped their own lives. Recognising their own social environments as both heritable and contributing to research-worthy health outcomes denoted a level of lived intimacy with the biosocial, extending it from the parameters of birth cohort analysis, to a fundamental and ordinary fact of participants’ lives. Exploring this along lines of intergenerational trauma, ALSPAC participant and adult daughter Lucy said the following:

Honestly like as a *teacher* for me I—I can see the effects of how a parent might be brought up and the things that have affected their life and how that then feeds into like the child? And I feel like a *multigenerational* study will be able to really put that into perspective.

Having one’s own multiple generations in the study clearly leads participants to think biosocially about the impacts of changing social environments on health and development. Indeed, some ALSPAC participants framed Children of the 90s as a positive tradition that is itself socially heritable. Original mother Sue said:

It means a lot to think that Jackie’s carried it on… cause some kids like [pauses] once, like they get older, it’s like, ‘Oh, I ain’t doing that, I don’t wanna do that,' and they don’t… involve their kids again, where she’s carried on and done what I’ve done with her, and it’s just really nice to see that.

Alice and her daughter Lauren also discussed how it felt normal and natural to continue in the study and to pass the experience onto Lauren’s child. Passing down the social world of participation was a motivating factor particularly when participants felt their families would benefit by their involvement, if not in biological, but in social ways. Original daughter Lucy, who had been involved her whole life, said, “I wanted to continue—and I wanted her [my daughter] to join in because of… just the *positive* effect I think it had on my life,” continuing that she had “fond memories… of being at Children of the 90 s…. everyone was so friendly… and they always fed you food which I liked… so I always found that… it was just a *happy* thing for me?” In turn, Lucy’s mother, Maeve, discussed a sense of safety and community that she gained from taking part with Lucy:

Part of the reason and I think why I stay involved [was that] … I always felt safe there, and it felt like we were contributing to something, because… my mum died when I was a child so my upbringing… it wasn’t particularly very happy… and, erm, when I had Lucy, you know, I definitely had the sense of I wanted… some safety, some continuity, and… all those things that people want [laughs] for their children… I think that I may have broken the cycles… And I think that’s what we hope the “Children of the 90s” [will do], it will come out with certain things that can help if people want it, to enhance their life, but also to break that cycle.

Interestingly, Maeve’s discussion of ‘broken cycles’ covertly points to the idea of inherited trauma, a widely circulated model of biosocial heritability which has gained particular traction in public discussion of epigenetics. Whilst it is unclear from Maeve’s comment whether she believes her participation directly benefits her daughter’s physiological health in an epigenetic way, it is clear that she spots an important link between socio-environmental upbringing and health. The safe and stable environment provided by ALSPAC offered a sense of community which seemed lacking in Maeve’s own past, and which she wanted to ‘hand down’ to her daughter in order to bestow safe communal spaces and happy childhood memories. In this way, cohort participation facilitates an intimate passing down of a social world whilst also affording the possibility for reversing cycles of adversity, not just for participating families, but for imagined beneficiaries of the research. Additionally, the notion of broken cycles speaks to the human potential for resilience and plasticity, concepts which invoke biosocial epistemology, and which Maeve saw play out through the social form of the study.

Whereas at ALSPAC participation can pass down to all children of study participants, no matter when they are born, Pelotas participants are often but not always guaranteed this. Original Pelotas participant Vivian had enrolled her young child simply because it was harmless, free to do, and beneficial for others, whilst another, Raquel, articulated she felt lucky her son happened to be born during recruitment years and that she hoped he would remain in it as an adult for many years to come. Carla, whose son was born outside the recruitment window and, therefore, was not enrolled in the study, despite her hoping he would have been, still felt that he may be getting indirect benefits because her continued contribution would help improve public health for future generations. For Pelotas participants, the social heritability of the study appeared less motivating than the safeguarding of biological health, with mothers such as Vivian commenting that outside of better health care access “we don’t really benefit from it”.

The social aspect of intergenerational participation in birth cohorts, however, often remains invisible. When framed as a mere externality of the data collection process, the intimate reality of participation cycles and their inherent biosociality is easily overlooked. We suggest, in contrast, that inheriting birth cohort participation is itself a biosocial process, one which prompts a reflexive examination of how the health issues faced by individuals and families evolve over time. Within the context of participation, emerging health issues and behaviours are established both by a changing medical climate, but also through each family’s corresponding practices and attitudes. A question emerges from these narratives, self-analyses, and social commentaries, as to what is legitimately considered by the research community as ‘biosocial knowledge’ and to what extent those who generate the data are able to lay a claim to the types of understandings which are subsequently made by participants.

### Articulating biosocial ‘expertise’: scientific ascendancy versus embodied knowledge

In this final section, we discuss two forms of ‘expertise’ within birth cohorts, the clinical/professional and the lived/embodied, both of which, we contend, need to be considered as relevant by biosocial researchers. As discussed above, research participants have a deeply intimate relationship with the process of generating biosocial knowledge. However, despite this level of comprehension, participants in our study repeatedly second guessed their intelligence and their overall grasp of cohort research outcomes. Below, we outline how birth cohort participants navigated this tension between the study professionals’ and their own ‘lay’ knowledge, to illustrate how everyday understandings of the biosocial exist in the space between scientific research and subjective experience. In spite of participants’ hesitancy to employ terms such as ‘epigenetic’ or claim academic expertise, biosocial knowledge is nonetheless present in their bodies and narratives. An example elaborated on from the previous section is how participants’ engagement with changing socio-temporal environments, for example with emerging dietary practices or differing uses of technology, provides anecdotal evidence of their lived intimacy with the biosocial and its impact on intergenerational health.

As previous sections have shown, participants from across ALSPAC and Pelotas had a fairly well-developed personal sense that both their biological and social data was being collected to capture a more holistic and comprehensive picture of human health. As Lauren, an original ALSPAC participant, said, using an intergenerational approach seemed “obvious” to the aims of the cohort, as it provided an important lens into how environmental changes over time impact peoples’ biologies. However, at ALSPAC in particular, participants were made self-conscious by their lack of scientific proficiency or clinical understanding and seemed curbed by their own sense of themselves as passive research subjects in contrast to ‘knowledgeable’ clinicians and researchers.

When reaching for exact or specific biosocial examples ALSPAC participants became notably hesitant, frequently caveating their answers with laughter or admitting “we don’t know” or “that’s probably wrong!” For both Alice and Maeve, participating grandmothers, their lack of confidence was demonstrated by their speech slowing down, trailing off, and wobbling with uncertainty as they attempted to contextualise experiences they’d had in the study (i.e. bone density scans) into a biosocial model. As Maeve suggested:

It’s about personality, it is about genetics… it’s about putting all that together [for example] ‘we think that these people could have heart disease earlier because of diet’… so that’s probably totally wrong! [laughs] but that’s what I see it as being [laughs].

Alice, too, was able to talk around the question in general terms but demonstrated uncertainty when trying to contextualise this into a practical example:

It is a very holistic approach, isn’t it? … medicine is becoming more and more holistic, and science now… [for example] whether psychology impacts on bone density. Obviously—well I was gonna say obviously it doesn’t, but maybe it does in some ways.

ALSPAC interviewees were more than happy to defer expertise to clinical researchers, as their sense of themselves as research subjects and their trust in the study led them to both expect and feel comfortable with a certain level of ignorance about the aims and results generated by the cohort. Alice, certainly, was delighted by the idea her information might be used to come up with things “we haven’t even begun to think of!” Her reluctance to pinpoint specific aims or outcomes was, therefore, compounded by the feeling that the studies’ purpose was to generate undetermined findings which wouldn’t necessarily be reflected by knowable aspects of her own life.

More subtle dynamics also existed around data collection which potentially increased the sense of a knowledge gap between ‘lay’ participants and ‘expert’ researchers. For birth cohort participants, the use of strange and modern cutting-edge technology, as described earlier, certainly added a layer of mystification to the data collection process. Conversely, the asking of certain ‘personal questions’ which participants couldn’t link to any obvious clinical value, somewhat confused them and led them to question their own understanding of the study. Some queried the seemingly scattergun nature of biosocial research which takes such a wealth of information that Lauren thought, at least some of it, must be “completely useless”. Elizabeth, who worked in a mental health hospital and had an interest in the clinical side of things, deliberated:

All like the medical stuff, and like you just think okay well this is like quite clearly—you can kind of see how it can be used? But some of the stuff I don’t know—I don’t know how it can be—and that—like you say in terms of like your personality traits and stuff, and—it says like how stressed are you, how much of the time? Or—bits and pieces—and I think that that’s harder to obviously analyse.

In Pelotas, original mother Beatriz, said that whilst she didn’t necessarily understand the overall aims of the study, she felt that she learnt a certain amount through being involved: “and then what you do, you’re learning too”. By attending closely to the sorts of data which are routinely collected, Vivian, similarly, constructed an image of what the study might be looking for. In the quote below, she noted how the biosocial practice of eating probably constituted valuable knowledge for public health:

Comparing there I believe the answers, the healthiest form perhaps of eating… a few years from now, you can get a sense that really, people who have had a healthier life, have lived longer, have, uh, like that, they didn’t have diseases, they didn’t have so many problems, you know?

Whilst a similar hesitancy existed among our Pelotas interlocutors about what the study was for— “I don’t know… I don’t know” —they were generally more forthcoming and confident in providing specific biosocial examples from their own lived experience. Original study participant Vivian situated these examples very much within her everyday life:

I believe that’s why… this research [is] referring to so many things… [because] not everything that is new is beneficial, right? … I used to hang out with friends, play, things like that… Today we can already see the harm it brings from staying for hours in front of a mobile phone screen, a computer screen… [imagine] what this does to a person’s brain, you know, imagine a child that’s developing, right?

Whilst ALSPAC participants also identified changes in diet and children’s use of technology as relevant biosocial data, they tended to bring these up in more general terms and less confidently in response to questions about the study’s scientific aims. It is possible that given a cultural context where social inequality is more visible, widespread, and openly acknowledged, Brazilian interlocutors found it easier to give examples of socially defined health disparities from their own lives, whereas English participants interviewed either didn’t experience these as members of the middle class, or if they identified as working class, were much shyer to discuss them. The topic of diet, for example, was mostly alluded to by ALSPAC interlocutors either in the abstract or, when discussed in relations to participants’ lives, tiptoed around as an issue. Maeve and her adult daughter Lucy made putative links between those on benefits, heart disease, and frozen pizza, whereas original mother Sue was the only ALSPAC participant to discuss the impacts of class on health in her own life, and even then, did so somewhat surreptitiously:

For instance [pauses] we’re kind of middle class, lower—not lower class but, do you know what I mean—where we don’t *struggle* struggle, but we’re not living—like you know… some weeks and that like we’ll have money to buy f—like quite a lot of food—and then another week, it’s a basic shop, do you know what I mean… but the thing is it could make a difference to people’s health… it does affect people’s health if there is something that they would be able to do about it.

In Brazil, mother and daughter Beatriz and Carla were more forthcoming in their discussion of class, diet, and food culture within their own homes, as personal intergenerational practices which constituted daily family life. Beatriz reflected on the importance of breastfeeding on infant development and the “more basic” foods she fed Carla in her childhood such as rice, beans, corn flour and sweet potatoes. Carla, too, remembered her mother giving them “lots of fruits, lots of vegetables,” although nowadays said she tends to “eat a lot of nonsense” because it is quicker and easier to prepare. Adult daughter Carla contextualised these private practices through her own class status as pertinent biosocial knowledge for the study:

Once I talked to a young man… over at the epidemiology centre where we participated [about]… people being poorer and not having the conditions… how are we going to say are being anaemic, or being, I think because being poor means not being not so well nourished… And the worst thing is, you know? We’re growing up, we’re running around, we’re eating more nonsense, we don’t have so much time, okay, we have to work every day, like it’s a rush to go one way and the other, and take turns, you know? We end up eating very little, and I think that’s a little bit of what the guy [researcher at the centre] wanted to talk about, you know?

Carla’s description of ‘running around’ and ‘eating nonsense’ demonstrates a lived somatic knowledge of the biosocial link between poverty, lack of time, malnutrition, and development. Her ability to give a more concrete biosocial example, in contrast to the relative vagueness of ALSPAC participations, perhaps owed to the corresponding prevalence of these issues in her family’s life, or her comparative openness to discuss them during interview. This subjectively derived understanding was then later confirmed during an interaction with one of the epidemiologists on site, illustrating how Carla’s participation in the study directly contributed to her own understanding of what the biosocial is. Discussions of poverty and nutrition, whilst clearly signalling epidemiological models such as the social determinants of health (Wilkinson 2003), were here articulated by participants as assemblages of social experiences, wherein the clinical distinctions between exposure variables were blurred into an overall sense of participants’ material entanglement with the world around them. Participants did not necessarily extract the social from the biological in their descriptions, but saw their mutuality as a fundamental truth likely to constitute the sort of information utilisable by researchers. The opportunities afforded by participation to reflect on the ever-changing nature of their lives, so thoroughly intimate, afforded an empirically produced biosocial knowledge which was no more, or less, remarkable than the unfolding nature of their intergenerational lives.

## Conclusion

This paper is situated in reaction to and in dialogue with the present “biosocial momentum” (Pálsson 2016) in life sciences research and our want to diversify the voices, knowledges, and narratives which constitute this burgeoning arena. The archetypal nature of ‘epigenetics’, as a stand-in for all things biosocial, has rightly warranted caution from the social science community for its overly simplistic, and sometimes scientifically inaccurate, public portrayal of the relationship between human ecologies and health, demanding a new and needed space for interdisciplinary research complexity. This is both a methodological and a moral issue. As Gísli Pálsson (2016) notes, new thinking around the biosocial must be cognisant of its tarnished past. The history of academic interest in this field bears the dark legacy of eugenics and racial determinism, supported by practices such as craniometry and, more recently, the use of nationally collected genetics databases to monitor, influence, and even control the state populace and a global image of the national body (ibid, p. 107). Such practices abound when discussions of biometric difference and variation categorise humans against each other, reflected in present day concerns that mothers are being over implicated by the epigenetic focus on the perinatal period (Lappé and Jeffries-Hein 2023), or that social determinants are being clustered to emphasise adversity rather than resilience (Filipe et al. 2021). Quoting Edwin Ardener’s (1989, p. 110) critique of ethnic categorisations within demography— “if numbers: counted by whom, and for whom?” —Pálsson (2016) reiterates the importance of obtaining multiple perspectives, including those ‘on-the-ground’, to construct a socially equitable scientific paradigm for the future.

Cognizant of these concerns and challenges, we have explored how research participants in birth cohort studies understand and make sense of biosocial science from the ‘bottom-up’. We found that, despite deferring expertise to clinical professionals, study participants demonstrated a clear understanding of biosocial entanglement and plasticity generated through contemplating their everyday experiences through the lens of specific research practices. In reflecting on their contributions, interlocutors drew heavily on their embodied experiences of participation as well as the intergenerationally captured dimensions of their lives. Discussing the study, both in terms of its mechanics and the data it seeks to capture, provided participants the opportunity to reflect on their own living histories, where “at all levels, the biological and the social are *in* one another” (Meloni et al. 2018, p. 5). Participants showed how biosocial knowledge, whilst remaining a conceptually challenging and methodologically contentious area within academia, is both an everyday and ordinary reality.

Our aim in communicating these findings is to recognise participants as co-producers of scientific knowledge, whose lived comprehension of the biosocial provides a grounding counterbalance to interdisciplinary tensions and reductive media depictions in this field. Recognising the intrinsic biosocial complexity of human life, as expressed in our interviews, demands caution against research models which simplify the relationship between biology and society, interdisciplinary models which anthropologist Elizabeth Roberts (2022) refers to as “causal enclosures” where social causes are ‘made small’ by biological reductionism (see also Neufcourt et al. 2022; Vineis and Barouki 2022), as well as pushing back against diminutive biosocial narratives in the public arena. Whilst our interlocutors repeatedly claimed scientific ignorance, they nonetheless communicated a powerful biosocial perspective which points to the value of co-produced perspectives.

The insights outlined in this article into participants’ biosocial reflections, knowledge-making, and already-held expertise would not be possible without the use of interactive qualitative methods, namely ethnographic interviews, to fully elucidate them. This highlights the relevance of, and ongoing need, for innovative qualitative methods in birth cohort studies and research which make participants’ voices and experiences visible (Watson et al. forthcoming). At the same time, the perspectives of the small number of intergenerational participants from both the cohort studies discussed in this article, provide novel possibilities for further comparative work on birth cohort participation across time and space in an unequal world. Our findings begin to suggest divergences, across the different global contexts of the UK and Brazil, between the relative motivations, comprehensions, and value of taking part in birth cohort research, which tentatively produce original and locally situated conceptions of the biosocial. In a higher-income context, the biosocial appears to emerge as a more exploratory, playful, and undefinable paradigm marked by novel technological advances, access to new social communities, and uncertain medical futures, whereas the comparative lower-income context seemingly generated a much more pragmatic and utilisable concept for ensuring better access to resources and improving overall population health. These divergences, whilst subtle, point to an underlying conception of the biosocial as an ‘opportunity for progress’, operationalised in divergent ways across two socio-cultural settings. Whilst this preliminary analysis is limited to the sample size of the study, we hope it opens the door to future explorations in this area. Additionally, participants’ expectations of what cohort participation can bring to them and their families in terms of knowledge and tangible benefits to their health and well-being were at times exceeded but also at times either not met or at risk of not being met in different ways for those in Brazil and the UK. It is important for the communities of researchers who design and maintain cohort studies to recognise that the diverse social world of participation for the everyday people who enrol is multifaceted, to recognise participants’ biosocial knowledge and expertise, and to continue to leverage it to propel research that is aligned with under-studied aspects of participants’ lives.

## Data Availability

The evidence on which this article is based is on qualitative data generated as part of a pilot study undertaken with the ALSPAC and Pelotas Cohort Study participants. This data set is not publicly available to ensure confidentiality and preserve anonymity of participants.

## Footnotes

1 The Biosocial Lives of Birth Cohorts project is a four-year project led by Professor Sahra Gibbon at University College London. It is funded by an Investigator Award in the Social Sciences granted by the Wellcome Trust.

2 Epigenetics is not the only field of biosocial science gaining traction. Human microbiome research, neuroscience are also subject of public and media discussion and representation of how the social shapes biologies.

3 This fieldwork in Brazil was carried out by Valéria Aydos and Helena Fietz. Due to Covid19 restrictions, it was done remotely.

4 The total sample size for analyses using any data collected after the age of seven is 15,447 pregnancies and 14,901 children.
